# Editorial: Catatonia across the lifespan

**DOI:** 10.3389/fpsyt.2022.1048715

**Published:** 2022-12-12

**Authors:** Jorge Cuevas-Esteban, Jordi Serra-Mestres, Sandeep Grover, Walter Jaimes-Albornoz

**Affiliations:** ^1^Hospital Germans Trias i Pujol, Badalona, Spain; ^2^Germans Trias i Pujol Health Science Research Institute (IGTP), Barcelona, Spain; ^3^Universitat Autònoma de Barcelona, Barcelona, Spain; ^4^Centro de Investigación Biomédica en Red de Salud Mental (CIBERSAM), Madrid, Spain; ^5^Cardinal Clinic, Windsor, United Kingdom; ^6^Post Graduate Institute of Medical Education and Research (PGIMER), Chandigarh, India; ^7^Donostia University Hospital, San Sebastian, Spain

**Keywords:** catatonia, cognition, neuropsychiatry, transcranial electric stimulation, elderly, child-age

A neuropsychiatric syndrome called Catatonia comprises motor, emotional, behavioral, and autonomic abnormalities. It is hence a psychomotor syndrome as it does not only affect motility. Therefore, it can be distinguished from other pure motor disorders such as extrapyramidal syndromes (1). Since the initial description of catatonia by Kahlbaum in 1874, it has also been connected to affective and medical conditions (2). The DSM-5 enhances diagnostic standards and the capacity to identify catatonia in the setting of any mental or medical condition (3).

Despite having a prominent clinical presentation and being a prevalent condition, catatonia is, nevertheless, underdiagnosed and undertreated. Its mean prevalence, regardless of the underlying condition, was 9.2% (4). Medical conditions have a higher prevalence rate (20.6%), followed by bipolar disorder (20.2%), postpartum psychosis (20%), autism (11.1%), schizophrenia (9.8%), and mixed psychiatric conditions (5.5%) (4).

Catatonia can occur at any stage in life linked to a wide range of physical and mental health conditions, having specific characteristics in children and in older adults (5). Between 0.6 and 1.7% of children and adolescents who are hospitalized for mental health pathologies have diagnosis of catatonia (6). It can occur in up to 37% of persons with NMDA receptor antibody encephalitis (7) and in 17% of patients with autistic spectrum disorders (8). Depending on the diagnostic criteria used, the frequency of catatonia in hospitalized psychiatric patients over the age of 65 ranges from 39.6 to 17.9% (9). In aged individuals, co-morbidity and neurodegeneration increase the likelihood of catatonic symptoms.

All this raises unresolved questions such as differences in the clinical presentation of catatonia, its symptomatic treatment, or the choice of the most appropriate screening instruments.

The Research Topic “Catatonia across the lifespan” aims to compile contributions reflecting new insights emerging from catatonia research across different age groups and populations. This Research Topic comprises 7 contributions; 4 original research articles, 1 systematic review, and 2 opinion articles. The manuscripts on this topic range from epidemiological, clinical, cognitive, to diagnostic and therapeutic aspects of catatonia in the different phases of life.

The correct diagnosis of catatonia remains a challenge for the clinician. Von Kanel et al. look into the connection between actigraphy-derived movement characteristics and symptoms of catatonia clinically evaluated. Contrary to clinical assessments, instrumental measurements lack observer bias, demand little training, and provide continual long-term assessments. The authors demonstrate how actigraphy can be used to measure particular catatonic symptoms including immobility/stupor and gazing. This might make it easier to detect catatonia, stage it, and monitoring catatonia in a therapeutic context. In another interesting paper in this Research Topic, Luccarelli et al., report the incidence of diagnosis, demographics, comorbidities, and inpatient procedures used in pediatric catatonic patients in general hospitals. The data extracted from their 900 patients generally agrees with the results of previous studies with much smaller populations. In this age group, catatonia is rarely diagnosed, it is more common in males and it is associated with significant and severe psychiatric and medical comorbidities. This severity increases significantly the cost of diagnosis and treatment, and this makes catatonia the most expensive pediatric mental health condition.

Serrat et al. undertook a systematic review of cognitive impairment in catatonic patients, analyzing evidence from 14 studies. The authors conclude that the most assessed cognitive domains on catatonia research are: attention, visuospatial and executive functions. These results, provide evidence supporting the frontal lobe syndrome theory proposed by Taylor (10).

Two opinion papers focus on challenges in the differential diagnosis: catatonia and dystonia, and catatonia and delirium. Ishizuka et al. propose focusing on molecular neurogenetic commonalities between catatonia and dystonia as a model to investigate the high co-morbidity of catatonia in patients with neurogenetic disorders. Tachibana et al. explain the difficulty of distinguishing delirium and catatonia, given their frequent overlap. They point out the impossibility of diagnosing both entities at the same time due to the DSM-5 diagnostic criteria. Finally, they discuss the therapeutic problems arising when there are doubts in the diagnosis.

Dawkins et al. investigated the psychopathological and phenomenological aspects of catatonia in a retrospective descriptive cross-sectional study, using information from a large dataset obtained from electronic healthcare records. Cluster analysis confirmed positive and negative symptom clusters, whilst component analysis revealed parakinetic, hypokinetic, and withdrawal components, all associated with diagnostic and prognostic variables. Fear was reported by a large minority of patients although narrative explanations were more common and varied.

Haroche et al., reported on 8 patients with catatonia treated with transcranial direct current stimulation (tDCS) targeting the left dorsolateral prefrontal cortex and temporoparietal junction, and who experienced significant symptomatic improvement. They also undertook a literature review on the topic, ascertaining 5 further cases treated with tDCS who had improved. The authors suggested that their results indicate that tDCS, in addition to pharmacotherapy, seems to effectively reduce catatonic symptoms, and conclude that the specific efficacy of tDCS in catatonia remains to be demonstrated in randomized controlled trials.

## Author contributions

All authors listed have made a substantial, direct, and intellectual contribution to the work and approved it for publication.
